# Mitochondrial genome of a medicinal beetle *Blaps rhynchopetera* (Coleoptera, Tenebrionidae) and phylogenetic analysis

**DOI:** 10.1080/23802359.2019.1640650

**Published:** 2019-07-16

**Authors:** Min Zhao, Zhao He, Cheng-Ye Wang, Long Sun, Ying Feng

**Affiliations:** Key Laboratory of Cultivating and Utilization of Resource Insects of State Forestry Administration, Research Institute of Resource Insects, Chinese Academy of Forestry, Kunming, P.R. China

**Keywords:** Mitogenome, *Blaps rhynchopetera*, medicinal beetle, phylogeny

## Abstract

*Blaps rhynchopetera* is a species of folk medicinal beetle that has been used for a long time in Southwest China. The complete mitogenome of the beetle (GenBank accession number MK854717) is 16,149 bp in size, including 13 protein-coding, 22 transfer RNAs, two ribosomal RNAs genes, and a noncoding D-loop region. The D-loop of 1255 bp length is located between rRNA-S and tRNA^Ile^. The overall base composition of *B. rhynchopetera* is 41.58% for A, 10.31% for G, 31.77% for T, and 16.34% for C, with a high AT bias of 73.35%. The present data could contribute to detailed phylogeographic analysis of this valuable medicinal insect.

*Blaps rhynchopetera* (Coleoptera, Tenebrionidae) is a species of folk medicinal insect. It is mainly distributed in Yunnan, Sichuan and Guizhou Province of China (Zhao et al. 2007). This beetle is popular as medicine of anti-inflammatory and analgesia in local, especially among the minority Yi people. Elucidating the structure and function of *B. rhynchopetera* mitogenome is important for understanding its diversity, genetics and evolution.

The specimen of *B. rhynchopetera* was obtained from Kunming, Yunnan, China (N 25°02′, E 102°42′) and deposited in the Insect Collection of Research Institute of Resource Insects with an accession number RIRI-w-20190418. Sequencing work of the complete mitogenome of *B. rhynchopetera* was performed by Illumina Nextseq500 in Beijing Microread Genetics Co., Ltd., with a total data volume 4G (150 bp Reads), and high-quality reads were assembled from scratch using IDBA-UD and SPAdes (Gurevich et al., 2013). Protein-coding genes (PCGs) of the *B. rhynchopetera* mitogenome were identified using BLAST search in NCBI, and tRNA genes were identified using the tRNAscan-SE search server (Schattner et al. 2005).

The gene order and orientation of *B. rhynchopetera* mitogenome are identical to the most common type of Coleoptera insects (Wang et al. 2016; Bai et al. 2018). It was 16,149 bp in size (GenBank accession number MK854717), including 13 typical invertebrate PCGs, 22 transfer RNA genes, two ribosomal RNA genes, and a noncoding control region (D-loop). The A + T content of the whole *B. rhynchopetera* mitogenome is 73.35%, showing an obvious AT mutation bias (Eyre-Walker 1997). The D-loop region exhibits the highest A + T content (78.59%) in the *B. rhynchopetera* mitogenome.

All PCGs use standard ATN as a start codon. As for the stop codon, 11 PCGs had the common stop codon TAA, while *COX3, ND4* terminated with incomplete stop codon T. Similar cases could be found in other insect mitogenomes (Yin et al. 2012). All the tRNAs except *tRNA^Ser^* (AGN) could be folded into the typical cloverleaf secondary structures. The unusual *tRNA^Ser^* (AGN) lacks dihydrouridine (DHU) arm.

Based on the concatenated 13 mitochondrial PCGs sequences of 12 species from Tenebrionidae, the neighbour-joining method was used to construct the phylogenetic relationship of *B. rhynchopetera* with 11 other Tenebrionidae beetles (Figure 1). *Blaps rhynchopetera* was clustered with *Platydema* sp. PLA01, indicating a close relationship between *Platydema* and *Blaps*. This mitogenome data might be also useful for further phylogeography analyses in Tenebrionidae.

**Figure 1.**
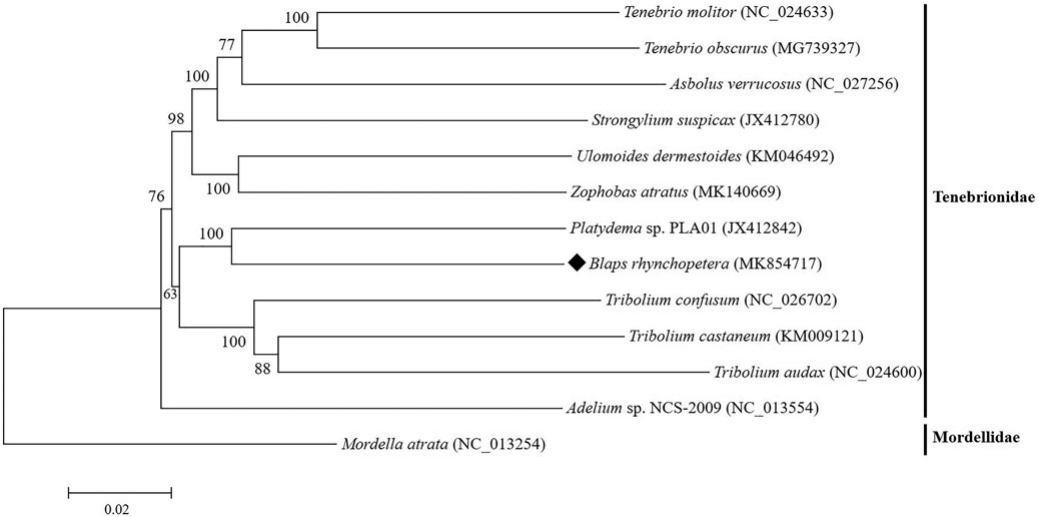
Phylogenetic tree showing the relationship between *B. rhynchopetera* and 11 other Tenebrionidae beetles based on neighbor-joining method. *Mordella atrata* (Mordellidae) was used as an outgroup. GenBank accession numbers of each species were listed in the tree.
